# Effect of *BF*839 + earthworm protein supplement on motor and some non-motor symptoms of Parkinson’s disease: a randomized clinical trial

**DOI:** 10.3389/fneur.2024.1371791

**Published:** 2024-09-06

**Authors:** Ting Zeng, Chuhui Lin, Yuhong Deng, Weiwen Zhu

**Affiliations:** ^1^Department of Clinical Nutrition, The Second Affiliated Hospital of Guangzhou Medical University, Guangzhou, China; ^2^Institute of Neuroscience and Department of Neurology, The Second Affiliated Hospital of Guangzhou Medical University, Guangzhou, China; ^3^Key Laboratory of Neurogenetics and Channelopathies of Guangdong Province and the Ministry of Education of China, Guangzhou, China

**Keywords:** *Bacteroides fragilis* 839, earthworm, gut microbiota, Parkinson’s disease, probiotics

## Abstract

**Introduction:**

Some studies have found that probiotics have the potential to treat PD, and earthworm protein is a traditional Chinese medicine used for the treatment of PD. The purpose of this study was to evaluate the safety and efficacy of *Bacteroides fragilis* 839 (*BF*839) + earthworm protein supplement as an adjunctive therapy for PD and to observe changes in the gut microbiota.

**Methods:**

Forty-six patients with PD were recruited for a 12-week 1:1 randomized, double-blind, placebo-controlled clinical trial to evaluate changes in motor and some non-motor symptom scores and detect metagenomic changes in the gut microbiota.

**Results:**

From baseline to 12 weeks, compared with placebo, the trial group showed significant reductions in the United Parkinson’s Disease Rate Scale (UPDRS) total score (−7.74 ± 5.92 vs. –1.83 ± 4.14, *p* < 0.001), UPDRS part I (−0.72 ± 0.81 vs. –0.20 ± 0.72, *p* = 0.026), UPDRS part II (−2.50 ± 2.24 vs. –0.22 ± 1.98, *p* = 0.001), UPDRS part III (−3.43 ± 3.42 vs. –1.33 ± 2.65, *p* = 0.024), and UPDRS part IV (−1.13 ± 1.19 vs. –0.15 ± 0.57, *p* = 0.001). Significant reductions in the Hamilton Depression Scale-24 score (−3.91 ± 3.99 vs. +1.15 ± 3.42, *p* < 0.001), Self-Rating Anxiety Scale scores (−7.04 ± 5.71 vs. –1.23 ± 2.34, *p* < 0.001), and Constipation scoring system scores (−8.59 ± 4.75 vs. 0.27 ± 1.24, *p* < 0.001), were also noted. In the trial group, one patient experienced mild eczema and one suffered low blood pressure, which could not be conclusively attributed to supplementation. Compared to the placebo group, the trial group showed a marked increase in *Enterococcus faecium* and a decrease in *Klebsiella.*

**Conclusion:**

This study is the first to report that probiotics plus earthworm protein can remarkably improve the motor and some non-motor symptoms of PD without serious adverse effects. However, further clinical trials and exploration of the underlying mechanisms are required.

**Clinical trial registration:**

Clinical trial registry http://www.chictr.org.cn/, Identification No: ChiCTR2000035122.

## 1 Introduction

Parkinson’s disease (PD) is a common neurodegenerative disorder. The primary treatment involves drugs such as levodopa to elevate dopamine levels and ameliorate symptoms. Currently, there are no preventive or disease modifying treatments for PD. As the disease progresses, the symptomatic treatment needs to be escalated and becomes more complex, which results in greater risk of side effects.

The relationship between PD etiology and gut microbiota imbalance has garnered increasing interest. PD has been proposed to originate in the gut (1). The gut microbiota composition differs between patients with PD and healthy individuals (2). Transplantation of feces from patients with PD into PD mice model aggravates motor deficits (3), and supplementation with mixed probiotics (4) slightly improves the movement dysfunction of PD mice model. Supplementation with a single (5) or mixed (6) probiotic improves non-motor symptoms such as gastrointestinal constipation, abdominal pain, bloating, anxiety, and sleep disturbance in humans. However, few studies have reported that probiotics can extensively improve motor function in patients with PD.

*Bacteroides fragilis* (*BF*) abundance in the feces of patients with PD is low (2). *BF* has attracted much attention in the second-generation probiotics studies (7). *BF839*—a non-toxic *BF* strain—is a symbiotic intestinal bacterium isolated from the feces of healthy infants (8). *BF839* prevents intestinal and respiratory diseases and promotes physical growth and development in children (9). We recently reported that *BF839* was effective in treating psoriatic disease (10), refractory epilepsy (11), and autoimmune epilepsy (12) in humans and improves learning and memory in mice with fragile X syndrome (13). Because it can substantially affect the brain, *BF839* may also play a role of PD treatment.

The traditional medicinal use of earthworms, also known as Lumbricus, in China dates back 2,000. Per an analysis of multiple classical prescriptions of Chinese traditional medicine for the treatment of PD, the usage frequencies of earthworms were 95% (14). Thus, earthworms are a “common drug” for treating PD in Chinese traditional medicine. Earthworm proteins are an approved new food resource in China and have been on the market for 13 years without serious adverse effects. Lumbricusin, an antimicrobial peptide isolated from earthworms, enhances neuroprotection and ameliorates motor dysfunction in a PD mice model (15).

This pilot study evaluates the safety and efficacy of *BF*839 + earthworm protein supplementation as an adjunctive therapy for patients with PD, compared with a placebo. The preliminary mechanism was explored by detecting metagenomic changes in the patient’s gut microbiota.

## 2 Patients and methods

### 2.1 Ethical considerations

This single-center, double-blind, randomized, placebo-controlled trial was conducted at the Neurology and Clinical Nutrition Department of the Second Affiliated Hospital of Guangzhou Medical University. The study was approved by the hospital’s Ethics Committee (Project No. 2019-hs-42), and is registered in the Chinese Clinical Trial Registry (http://www.chictr.org.cn/; Identification No: ChiCTR2000035122). Participants provided written informed consent before starting the trial.

### 2.2 Trial design and participants

**Figure 1:**
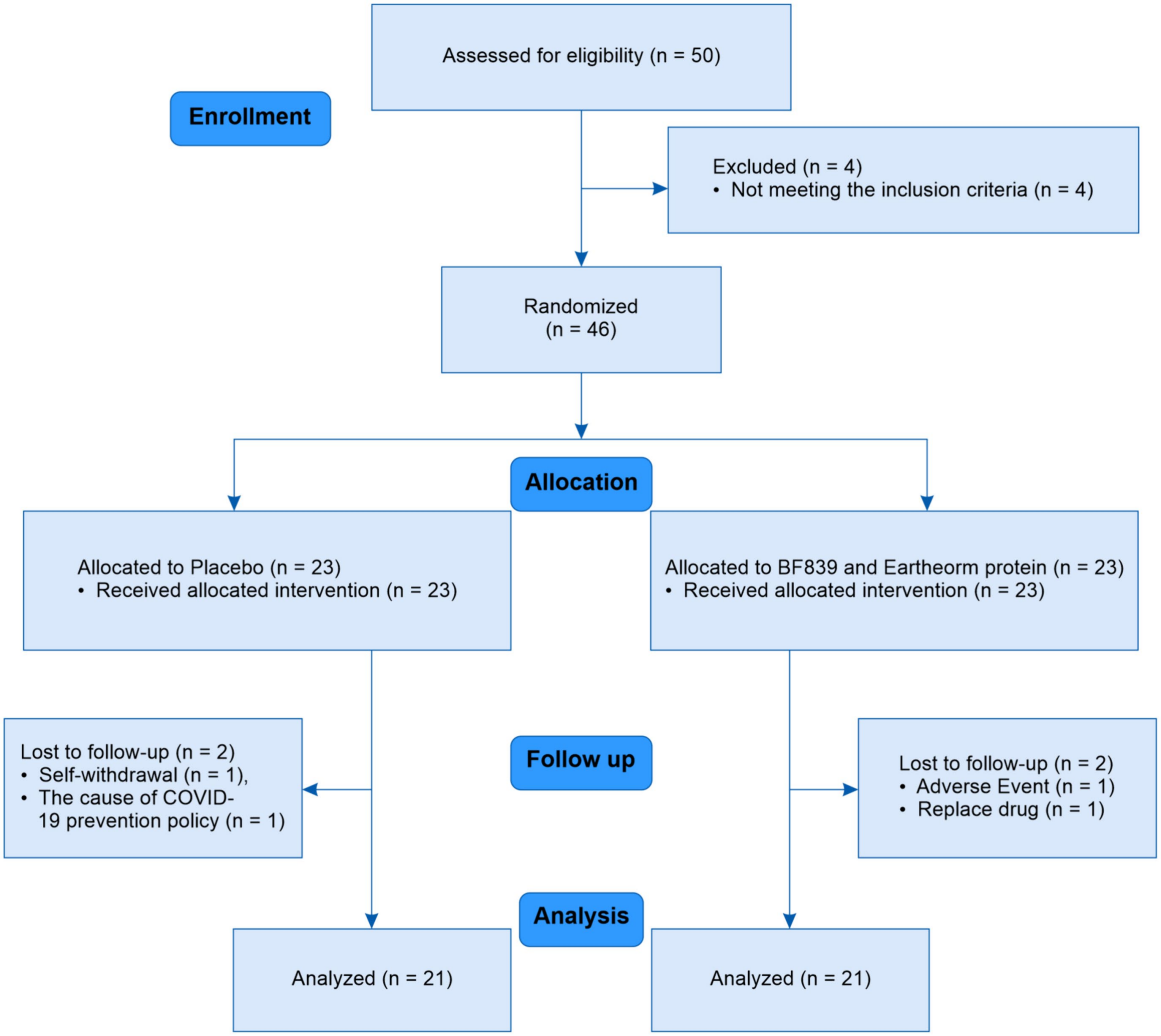
Trial process.

A 12-week Randomized Controlled Trial (RCT) was performed, and metagenomic changes in the gut microbiota of some patients were detected. Figure 1 illustrates the trial process. Participants were enrolled from September 1, 2020 to August 31, 2021. The inclusion criteria were: (1) meeting the diagnostic criteria of the Movement Disorder Society (MDS) Clinical Diagnostic Criteria and Hoehn and Yahr stages 1–3 (16, 17), indicating clinically established PD; (2) the ability to independently or with assistance from family members complete examinations, questionnaires, and provide medical history; (3) use of anti-parkinsonian drugs such as levodopa, dopadecarboxylase-inhibitor, dopamine agonists (DA), monoamine oxidase type B inhibitor, catechol-*o*-methyltransferase inhibitor, amantadine hydrochloride, and trihexyphenidyl provided that the dosage remained unchanged in the 30 days leading up to enrollment. The exclusion criteria were: (1) severe cognitive impairment or aphasia and dysarthria leading to communication difficulties; (2) severe liver or kidney dysfunction or tumors; (3) use of immunosuppressants, antibiotics, or other probiotics, or fecal microbiotic transplantation 30 days prior to enrollment; (4) history of severe and uncontrolled hypertension; and (5) spontaneous bleeding, coagulopathy, or long-term use of anticoagulants. The exit criteria were: (1) lost to follow-up; (2) unacceptable or serious adverse events; (3) adjustment of anti-parkinsonian drug dosage during the trial; and (4) using <20% of the trial supplement dose. All patients had primary PD without family history of PD. However, genetic testing was not performed, and the exclusion of patients with multifactorial PD was based primarily on the absence of established concomitant neurologic conditions, such as dementia, brain atrophy, stroke.

An independent neurologist is responsible for the assessment of patients. The assessment scales include the UPDRS scale, Hamilton Depression Scale-24 (HAMD-24), Self-Rating Anxiety Scale (SAS), Constipation Scoring System Scale (CSS), and Minimum Mental State Examination (MMSE). The patients were evaluated at day 0, 6 weeks and 12 weeks. Each evaluation was performed at the same time of day for each patient before and after enrollment, thus basically excluding the influence of motor fluctuations and dyskinesia on the results. In fact, four patients in this trial experienced motor fluctuations, yet they were in the ‘ON’ state during each evaluation. The response rate was defined as the proportion of Minimal Clinically Important Difference change in patient scores from baseline to 12 weeks (Supplementary Data 1.1).

### 2.3 Sample size estimation

Per previous study results (18), the total UPDRS reduction from baseline with placebo as adjunctive therapy was 8.5% in Hoehn-Yahr I–III patients. In our preclinical trial data, we observed a substantial decrease of 45% in the UPDRS. We set the alpha value at 0.05, power effect value at 80%, and a ratio of 1, and calculated the required sample size to be 40 (20 per group) using pass 11.0 software (NCSS, Inc., USA). Considering a 15% dropout rate, 46 patients (23 patients per group) were enrolled.

### 2.4 Recruitment and randomization

Participants were allocated (1:1) to the trail or placebo group using a concealed random allocation from a computer-generated random numbers table produced using the Python programming environment. A nurse dispensed the trial supplement, but she did not know which trial supplement the patient received. *BF*839 + earthworm protein supplements and placebo were prepared and packaged by Guangzhou Totem Life Medical Research Co. Ltd., China. The two products are almost identical and cannot be distinguished. To ensure the authenticity of the statistical results, both the participants and researchers (including people of trial organization, follow-up, evaluation, data entry and statisticians) were blinded. Unblinding was only indicated upon serious adverse events, with approval of a Steering.

### 2.5 Interventions

10 g *BF*839 + earthworm protein supplement trial solution dissolved in 200 mL water, twice daily, or matching placebo as an adjunctive therapy for 12 weeks. 10 g trial supplement contains 10^6^ *BF*839 and 0.3 g earthworm protein. More details about *BF*839 and earthworm protein production can be seen in Supplementary material 1.2. The placebo was made from maltodextrin and had a similar odor and taste than did the *BF*839 + earthworm protein supplement with identical packaging. Concomitant anti-parkinsonian medications were retained, and no changes were allowed throughout the trial.

### 2.6 Adverse events

Patients were monitored for gastrointrial inal-related diseases, including common adverse events such as nausea, vomiting, diarrhea, constipation, increased exhaust, and rash. Unexpected adverse events were also recorded.

### 2.7 Stool sample collection and processing and analysis

Stool samples were collected from 20 randomly selected patients on both day 0 and week 12. Ten patients were selected from the placebo group; two failed to provide stool samples at week 12, resulting in a total of 18 samples. In the test group, 10 patients were selected, resulting in a total of 20 samples. Samples were collected at home and shipped to Shenzhen 01 Life Institute Co. Ltd. at room temperature for testing. R3.6.3 was used to calculate species count and Shannon diversity to assess microbiota diversity. Bray-Curtis distance and PCoA were applied to analyze microbiota composition changes, with a permutational multivariate analysis of variance performed to identify temporal and group differences in microbiota composition. Gene set alignment and intestinal metabolic modules prediction were performed (Supplementary material 1.3–1.8).

### 2.8 Statistical analyses

Clinical data were analyzed using per-protocol and intention-to-treat analyses. Statistical analyses were performed using SPSS statistical software (version 22.0; SPSS Inc., Chicago, IL, USA). Measurement data were expressed as mean ± SD, and the comparison between groups was analyzed using t-test. Count data are expressed as number of cases (%), and the chi-square or Fisher’s exact test was used for between-group comparison. Statistical significance was set at *p* < 0.05.

## 3 Results

### 3.1 Baseline patient characteristics

Of 50 patients screened between September 1, 2020, and August 31, 2021, 46 were included. In the trial group, one patient exited because of a change in anti-parkinsonian drugs, and another because of an adverse event. In the placebo group, one patient withdrew consent because he participated in another trial and another could not return to the hospital due to the COVID-19 prevention policy. Forty-two patients completed the trial. Table 1 presents their baseline characteristics.

**Table 1:** Patients’ baseline characteristics.

Basic information	Trial (*n* = 21)	Placebo (*n* = 21)	*P*
Sex			0.747
Male, *N* (%)	13 (61.9)	14 (66.7)	
Female, *N* (%)	8 (38.1)	7 (33.3)	
Age (year, mean ± sd)	62.29 ± 8.90	59.57 ± 7.77	0.299
Total UPDRS	22.33 ± 10.09	20.52 ± 7.21	0.614
Course of the disease(year, mean ± sd)	6.19 ± 4.33	6.14 ± 4.73	0.755
Hoehn-Yahr Disease stage			0.757
I-II installment, *N* (%)	12 (57.1)	11 (52.4)	
III installment, *N* (%)	9 (42.9)	10 (47.6)	
On medication(species, mean ± sd)	3.00 ± 1.05	3.01 ± 1.32	0.898
Levodopa + DDC-I, *N* (%)	19 (90.47)	17 (80.95)	0.378
Levodopa + DDC-I sustained-release tablets, *N* (%)	6 (28.57)	4 (19.05)	0.469
DA, *N* (%)	13 (61.90)	16 (76.19)	0.063
MAO-BI, *N* (%)	7 (33.33)	9 (42.86)	0.525
Artane, *N* (%)	2 (9.52)	3 (14.29)	0.634
Amantadine, *N* (%)	2 (9.52)	1 (4.76)	0.549

P, comparison between trail and placebo groups using chi-square test; DDC-I, dopadecarboxylase-inhibitor, dopamine agonists; MAO-BI, monoamine oxidase type B inhibitor.

### 3.2 *BF*839 + earthworm protein remarkably improved PD-related motor and non-motor symptoms

At 6 weeks, in the intention-to-treat analysis, the trial group exhibited a significant reduction in the UPDRS total and UPDRS part IV scores compared with the placebo group. Additionally, the trial group showed a non-significant downward trend in the UPDRS part I, II, and III scores than the placebo group. Compared with the placebo group, the trial group exhibited significant reduction in the HAMD-24, SAS, and CSS scores. A similar trend was observed in the per-protocol analysis (Table 2).

**Table 2:** Comparison of the scores of 0d, 6-week data with the baseline (day 0), and 12w-0d between the trail and placebo groups.

Scale score	Per-protocol analysis									Intention-to-treat analysis								
	0d			6-week data with the baseline (day 0)			12 weeks with the baseline (day 0)			0d			6-week data with baseline data (day 0)			12 week data with baseline data (day 0)		
	Trial (*n* = 21)	Placebo (*n* = 21)	*P^a^*	Trial (*n* = 21)	Placebo (*n* = 21)	*P^b^*	Trial (*n* = 21)	Placebo (*n* = 21)	*P^c^*	Trial (*n* = 23)	Placebo (n = 23)	*P^a^*	Trial (*n* = 23)	Placebo (*n* = 23)	*P^b^*	Trial (*n* = 23)	Placebo (*n* = 23)	*P^c^*
Total UPDRS	22.33 ± 10.09	20.52 ± 7.21	0.614	−4.30 ± 3.64	−1.83 ± 3.58	0.032	−8.37 ± 5.65	−2.00 ± 4.30	<0.001	21.54 ± 9.99	19.87 ± 7.21	0.653	−3.93 ± 3.69	−1.67 ± 3.45	0.037	−7.74 ± 5.92	−1.83 ± 4.14	<0.001
UPDRS part I mentation, behavior and mood	1.19 ± 1.17	0.52 ± 0.81	0.052	−0.45 ± 0.79	−0.24 ± 0.62	0.285	−0.79 ± 0.82	−0.21 ± 0.75	0.009	1.11 ± 1.15	0.48 ± 0.79	0.0035	−0.41 ± 0.76	−0.22 ± 0.60	0.339	−0.72 ± 0.81	−0.20 ± 0.72	0.026
UPDRS part II activities of daily living	8.30 ± 4.33	8.35 ± 3.63	0.536	−1.38 ± 2.16	−0.33 ± 1.31	0.064	−2.74 ± 2.20	−0.24 ± 2.07	<0.001	8.28 ± 4.17	8.15 ± 3.53	0.909	−1.26 ± 2.09	−0.30 ± 1.25	0.067	−2.50 ± 2.24	−0.22 ± 1.98	0.001
UPDRS part III motor examination	10.45 ± 4.52	10.69 ± 4.10	0.859	−1.98 ± 2.22	−1.11 ± 2.54	0.251	−3.76 ± 3.40	−1.45 ± 2.74	0.020	9.91 ± 4.68	10.35 ± 4.08	0.739	−1.80 ± 2.20	−1.02 ± 2.45	0.259	−3.43 ± 3.42	−1.33 ± 2.65	0.024
UPDRS part III speed, Facial expression	1.36 ± 0.94	1.05 ± 1.00	0.225	−0.09 ± 0.68	−0.19 ± 0.46	0.408	−0.45 ± 0.93	−0.12 ± 0.67	0.189	1.33 ± 0.90	1.04 ± 0.95	0.307	−0.08 ± 0.65	−0.17 ± 0.44	0.599	−0.41 ± 0.90	−0.11 ± 0.64	0.193
UPDRS part III Tremor at rest, action or Postural Tremor of hands	0.93 ± 1.12	1.55 ± 0.84	0.028	−0.14 ± 0.32	−0.21 ± 0.62	0.569	−0.29 ± 0.70	−0.26 ± 0.66	0.860	0.89 ± 1.09	1.46 ± 0.86	0.057	−0.13 ± 0.31	−0.20 ± 0.60	0.645	−0.26 ± 0.67	−0.24 ± 0.63	0.911
UPDRS part III Rigidity	1.02 ± 0.68	1.29 ± 0.92	0.487	−0.24 ± 0.45	−0.31 ± 0.68	0.904	−0.43 ± 0.51	−0.36 ± 0.71	0.840	198 ± 0.68	1.22 ± 0.91	0.320	−0.22 ± 0.45	−0.28 ± 0.65	0.695	−0.39 ± 0.50	−0.32 ± 0.71	0.714
UPDRS part III Figer Taps, Hand movements, Rapid Alternating Movements of Hands	2.12 ± 1.64	3.26 ± 1.64	0.076	−0.36 ± 0.96	−0.10 ± 1.06	0.458	−0.74 ± 1.37	−0.52 ± 1.44	0.740	2.07 ± 1.58	3.11 ± 2.00	0.057	−0.33 ± 0.92	−0.09 ± 1.01	0.406	−0. 67 ± 1.32	−0.48 ± 1.38	0.625
UPDRS part III Leg agility, Arising from Chair	1.29 ± 1.27	1.14 ± 0.99	0.907	−0.17 ± 0.76	0.00 ± 0.98	0.154	−0.55 ± 0.93	0.07 ± 1.04	0.030	1.26 ± 1.21	1.13 ± 0.94	0.686	−0.15 ± 0.73	0.00 ± 0.94	0.543	−0.50 ± 0.90	0.07 ± 0.99	0.050
UPDRS part III Posture, Gait, Postural Stablility, Body Bradykinesia and Hypokinesia	3.33 ± 2.63	2.73 ± 1.80	0.630	−0.55 ± 1.09	−0.57 ± 1.06	0.804	−0.98 ± 1.44	−0.67 ± 1.58	0.576	3.17 ± 2.57	2.63 ± 1.76	0.408	−0.50 ± 1.06	−0.52 ± 1.03	0.944	−0.89 ± 1.40	−0.61 ± 1.52	0.515
UPDRS part IV DYSKINESIAS	2.43 ± 2.64	0.93 ± 1.50	0.010	−0.50 ± 0.55	−0.071 ± 0.58	0.031	−1.23 ± 1.19	−0.17 ± 0.60	<0.001	2.28 ± 2.57	0.87 ± 1.44	0.026	−0.46 ± 0.54	−0.07 ± 0.55	0.019	−1.13 ± 1.19	−0.15 ± 0.57	0.001
MMSE	29.00 ± 1.58	28.86 ± 2.26	0.787	+0.10 ± 0.77	+0.43 ± 1.363	0.558	+0.43 ± 1.21	+0.62 ± 1.47	0.940	28.87 ± 1.63	28.91 ± 2.17	0.939	+0.09 ± 0.73	+0.39 ± 1.30	0.335	+0.39 ± 1.58	+0.57 ± 1.40	0.650
HAMD-24	14.19 ± 6.33	14.19 ± 7.61	1.000	−2.79 ± 4.71	+0.48 ± 2.22	0.032	−4.29 ± 3.98	+1.26 ± 3.57	<0.001	14.00 ± 6.13	13.96 ± 7.31	0.983	−2.54 ± 4.56	+0.43 ± 2.13	0.007	−3.91 ± 3.99	+1.15 ± 3.42	<0.001
SAS	43.51 ± 8.27	37.60 ± 6.57	0.014	−4.52 ± 4.58	−0.05 ± 3.31	0.001	−7.71 ± 5.51	−1.23 ± 2.43	<0.001	42.77 ± 8.40	37.59 ± 6.31	0.022	−4.13 ± 4.56	−0.04 ± 3.15	0.001	−7.04 ± 5.71	−1.23 ± 2.34	<0.001
CSS	16.15 ± 4.80	15.63 ± 3.56	0.705	−7.05 ± 2.78	0.26 ± 1.19	<0.001	−9.45 ± 4.05	0.42 ± 1.21	<0.001	16.09 ± 4.58	15.55 ± 3.32	0.705	−6.41 ± 3.36	0.18 ± 1.14	<0.001	−8.59 ± 4.75	0.27 ± 1.24	<0.001

*P*^a^: Comparison at baseline between trail and placebo groups. *P*^b^: Comparison at 6-week data with the baseline (day 0) between trail and placebo groups. *P*^c^: Comparison at 12 weeks with the baseline (day 0) between trail and placebo groups.

At 12 weeks, in the intention-to-treat analysis, the trial group demonstrated significant decreases in the UPDRS total, UPDRS part I, UPDRS part II, UPDRS part III, and UPDRS part IV scores compared to the placebo group. The trial group showed better improvement than the placebo group, especially in the leg symptom (Leg agility、Arising from Chair) of UPDRS part III score. Other symptoms assessed by UPDRS part III (Table 2), also showed a non-significant downward trend in the trial group compared to the placebo group. Compared to the placebo group, the trial group showed significant decreases in the HAMD-24, SAS, and CSS scores. A similar trend was observed in the per-protocol analysis (Table 2).

In intention-to-treat analysis n, at 12 weeks, compared with the placebo group, the trial group had a significantly higher response rate in patients with score decrease of total UPDRS≥8 point (47.82% *Vs* 4.34%, *p* = 0.001), UPDRS part II ≥ 2 point (60.87% *Vs* 17.39%, *p* = 0.003), UPDRS part III ≥ 5 point (30.43% *Vs* 4.34%, *p* = 0.02), and CSS >30% (95.65% *Vs* 0%, *p* < 0.001). Per-protocol analysis showed similar results (Supplementary Table S1). Two special cases in the trial group did not receive levodopa and took only rasagiline (1 mg/day). Their UPDRS scores reduced substantially from 13 and 14 at baseline to 5.5 and 4.5 at 12 weeks, respectively. Both patients regained their sense of smell at 12 weeks. Additionally, one patient in the trial group reported regaining his sense of smell.

### 3.3 Adverse events

In the trial group, 4.3% (1 of 23) of the patients reported mild eczema at 3 weeks, which resolved spontaneously after one week and did not lead to an exit from the study. Additionally, 4.3% (1 of 23) of the patients in the trial group experienced dizziness and low blood pressure (80–105 mmHg/50–60 mmHg), resulting in withdrawal from the study. However, this patient was concurrently using other Chinese medicines, including sodium aescinate tablets, ginkgo ketone drop pills, and compound Xueshuantong capsules containing notoginseng, *Astragalus*, *Salvia miltiorrhizae*, and *Radix scungshentch*, to improve circulation. Therefore, it is difficult to determine the correlation between these adverse events and the use of the trail medication. Additionally, two cases of bloating were reported in the placebo group; however, these did not meet the criteria for study withdrawal.

### 3.4 Changes in gut microbiota

**Figure 2:**
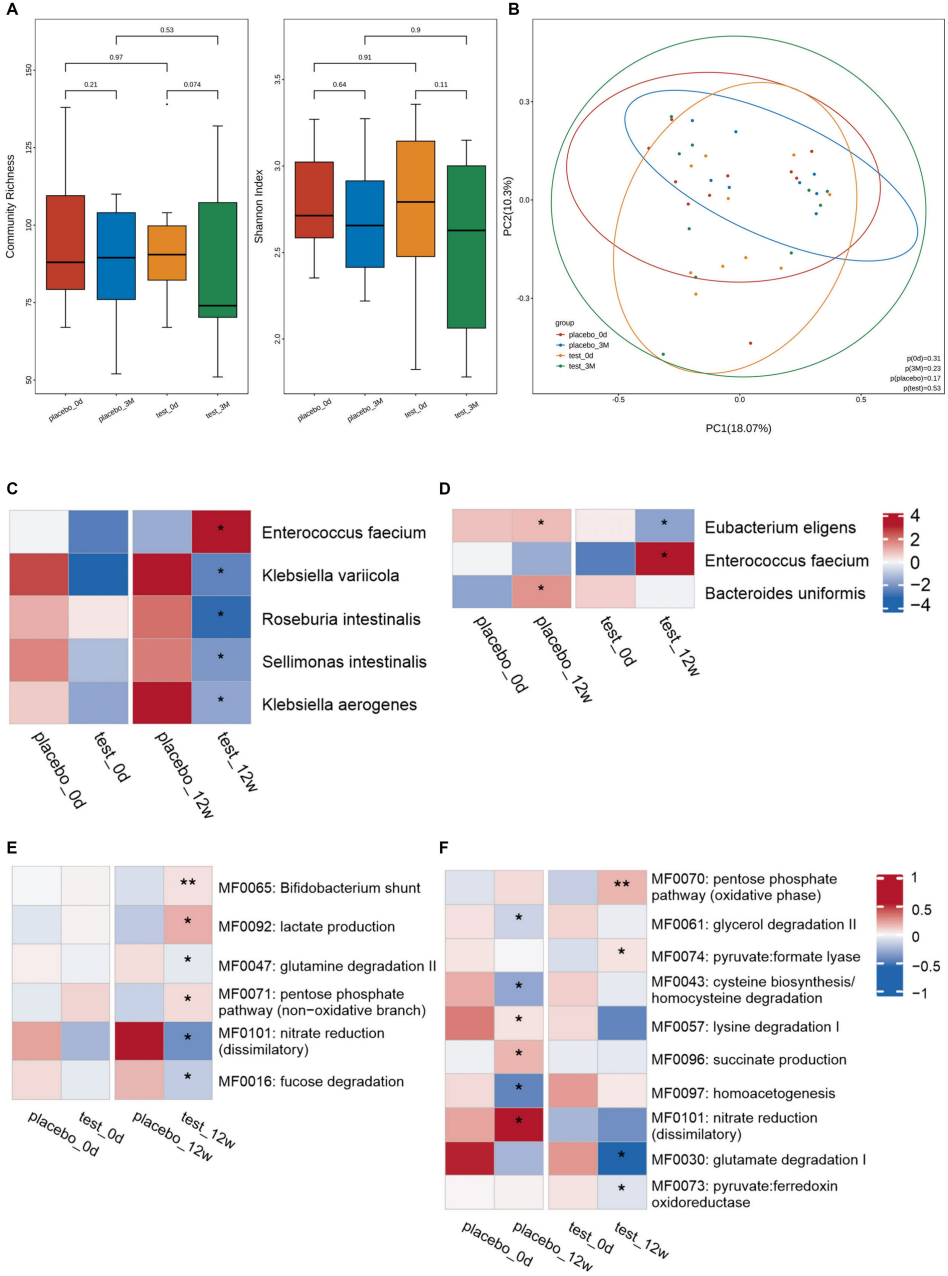
Changes in the intestinal microbiota. Diversity of microorganisms at the species-level: **(A)** Diversity of fecal microbiota at the species-level between the placebo and trial groups on day 0 and week 12: (left) community abundance and (right) Shannon diversity index. Elements of box plot: Centerline: median; end of the box: upper and lower quartiles; dot: outlier. **(B)** PCoA scores for the placebo and trial groups on day 0 and week 12 with different colors for each group of samples. Between-group differences in microbiota at the species-level: **(C)** Between-group comparisons conducted to assess the microbial abundance on day 0 and at week 12. No significant difference in microbial abundance was observed between the two groups at day 0. However, a significant increase/decrease in microbial abundance was observed between the two groups at 12 weeks. **(D)** Within-group comparison of the abundance between day 0 and week 12. At week 12, significant changes in abundance, including both increases and decreases, were observed in both groups compared to day 0. *p < 0.05. Analysis of the intestinal metabolic modules (GMMs): **(E)** Between-group comparison of the distribution of intestinal metabolic modules on day 0 and at week 12. A significant between-group difference was observed at week 12, but not on day 0. **(F)** Within-group comparison of the distribution of intestinal metabolic modules on day 0 and at week 12. Significant increases/decreases in abundance in both groups were observed at week 12 compared with day 0. *p < 0.05, **p < 0.01.

There was no significant difference in diversity (Figures 2A,B) and species-level abundance (Figures 2C,D) between the placebo and trial groups on day 0.

At 12 weeks, there was a greater decrease in microbiome diversity in the trail than placebo group; however, the difference was not significant (Figure 2A). No clustering was observed for the Bray–Curtis dissimilarity in principal coordinates analysis (PCoA) (Figure 2B).

Compared with the placebo group, the trial group exhibited a marked increase in the abundance of *Enterococcus faecium* at 12 weeks. Conversely, the abundances of *Klebsiella variicola*, *Roseburia intestinalis*, *Sellimonas intestinalis*, and *Klebsiella aerogenes* considerably decreased in the trial group (Figure 2C). When compared with day 0, the placebo group showed higher levels of *Eubacterium eligens* and *Bacteroides uniformis* at week 12. However, the trial group exhibited a higher abundance of *Enterococcus faecium* and a lower abundance of *Eubacterium eligens* at week 12 compared to day 0 (Figure 2D).

The metabolic functions of the neuroactive compounds encoded by gut microbes were analyzed. No significant differences were observed between the placebo and trial groups on day 0 and at 12 weeks. The bifidobacterial shunt, lactate production, and pentose phosphate pathway (non-oxidative branch) metabolic functions in the trial group were remarkably increased compared to those in the placebo group. Meanwhile, glutamine degradation II, nitrate reduction (dissimilatory), and fucose degradation considerably decreased (Figure 2E). At 12 weeks, succinate production, nitrate reduction (dissimilatory), and lysine degradation I-related functions were markedly enhanced in the placebo group compared to those at day 0. Contrastingly, glycerol degradation II, cysteine biosynthesis/homocysteine degradation, and homoacetogenesis considerably decreased. At 12 weeks, in the trial group, the pentose phosphate pathway (oxidative phase) and pyruvate: formate lyase increased compared to those at day 0. Meanwhile, glutamate degradation I and pyruvate: ferredoxin oxidoreductase substantially decreased (Figure 2F).

## 4 Discussion

We observed a significant diminishment of total UPDRS score (−7.74 ± 5.92 vs. –1.83 ± 4.14, *p* < 0.001), Hamilton Depression Scale-24 score (−3.91 ± 3.99 vs. +1.15 ± 3.42, *p* < 0.001), Self-Rating Anxiety Scale scores (−7.04 ± 5.71 vs. –1.23 ± 2.34, *p* < 0.001), and Constipation scoring system scores (−8.59 ± 4.75 vs. 0.27 ± 1.24, *p* < 0.001) when compared with the placebo group, which means implementing probiotics combined with earthworm protein intervention as an adjunctive therapy for PD patients were beneficial for both motor and certain non-motor symptoms. This improvement surpasses previous research findings (19), which reported a 6% decrease in the MDS-UPDRS total score with mixed probiotics, as our research revealed approximately a 35% (7.74/22.33) reduction (Table 2).

Several animal studies have shown that intestinal dysbiosis affects the occurrence and development of PD by increasing intestinal permeability, neuroinflammation, accumulation of abnormal levels of synuclein fibrils, oxidative stress, and production of neurotransmitters (20). However, the exact mechanism by which gut microbiota affects PD remains unclear. Non-toxic forms of *BF* can prevent intestinal inflammation in animal models of colitis, protects against experimental autoimmune encephalomyelitis, and activates intestinal sensory neurons (21). The administration of *BF* to offspring mice with maternal immune activation (MIA) and autistic traits has been shown to correct intestinal permeability by ameliorating MIA-related alterations in the expression of colonic tight junction proteins, specifically claudin 8 and claudin 15. This, in turn, corrects MIA-induced abnormalities in serum metabolites, including 4-ethylene phenyl sulfate, and improves communication, repetitive behaviors, anxiety-like behaviors, and sensorimotor function (22). While acknowledging the significant differences between humans and mice, the observed therapeutic effects may indicate that this could be one of the mechanisms underlying the efficacy of *BF* treatment, necessitating further research. Notably, despite the absence of detectable *BF* in the fecal samples of offspring treated with *BF* and no significant difference in microbial richness indicated by the PCoA score, *BF* supplementation still alleviated the changes in specific microbiota associated with MIA (22), this findings of Hsiao are consistent with our findings that *BF*839 was not detected in feces and that microbial community diversity, including diversity index and PCoA score, was not significantly different from that in the placebo group.

Our findings further support the notion that *BF* may not establish long-term colonization but can modulate the bacterial species associated with the disease (22), thereby improving symptoms. We observed a marked increase in *Enterococcus faecium* and a marked decrease in *Klebsiella faecium* in the trial group compared to the placebo group. These findings are consistent with previous studies, in which *Enterococcus faecium* was transplanted into mice with PD. The Enterococcus faecium significantly increased dopamine levels in the brain and ameliorated PD manifestations in these mice (23), while *Klebsiella* has been positively correlated with PD duration and severity (24), suggesting a mechanistic link. *Klebsiella* may produce metabolites that are toxic to dopaminergic neurons, directly damaging nervous system function; *Klebsiella* may trigger systemic inflammatory and immune responses by activating the intestinal immune system, affecting central nervous system function. The microbiome interacts with the brain through a complex network of pathways involving the immune system, vagus nerve, enteric nervous system, tryptophan metabolism, branched-chain amino acids, short-chain fatty acids, peptidoglycans, other microbial metabolites (25), and unidentified substances. Pinpointing the specific mechanism by which *BF*839 exerts its beneficial effects against PD is challenging, as it likely involves a combination of factors.

Although earthworms are one of the most commonly used anti-PD drugs in traditional Chinese medicine, the underlying mechanism remains unclear. To survive, earthworms must code and synthesize special proteins, especially those of the proteolytic isozyme family, in their digestive system. Earthworm proteases have been used to treat cerebrovascular diseases because of their fibrinolytic and anticoagulant effects (26). Patients with PD exhibit extensive or focal hypoperfusion of the cerebral blood (27). Probiotics may influence PD through diverse pathways, primarily by modulating metabolites, neurotransmitters, and nutrients that have the potential to reach the brain through the bloodstream. As a result, we hypothesized that enhancing cerebral blood flow perfusion could potentially increase the efficacy of probiotics. Lumbricusin, an 11-merantibacterial peptide (NH2-RNRRWCIDQQA) isolated from earthworms, significantly increases the proliferation of mouse neural stem cells (MNSCs) isolated from the mouse brain, enhances proteasome-mediated p27Kip1 degradation in MNSCs, protects MNSCs against 6-hydroxydopamine-induced apoptosis, and attenuates motor impairments in the PD mouse model (15). Therefore, Lumbricusin’s potential for treating PD is supported by our study. However, this disease modifying mechanism cannot explain completely the symptomatic improvement observed in the trial. The potential beneficial effect of the investigational product being strictly symptomatic.

The two patients who did not receive levodopa in our study had a greater improvement in UPDRS scores than patients who received levodopa. This is consistent with previous findings showing greater improvement in the UPDRS scores of patients taking pramipexole without concomitant levodopa (28). Therefore, patients may benefit more from earlier treatment without madopar. Surprisingly, three patients reported recovery of their lost sense of smell. Given that there is no effective method to improve the sense of smell in patients with PD, this study provides important clues for improving this symptom.

While probiotic microbial supplementation in humans is generally regarded as safe, it may cause an enhanced response to allergens during immune regulation. The occurrence of mild eczema in 4.3% of the patients in our study serves as evidence of this phenomenon. However, the severity and incidence of such reactions need to be further observed in larger sample sizes in future studies. Some studies reported that the earthworm protein capsule exhibited antihypertensive effects on spontaneously hypertensive rats by inhibiting Renin-angiotensin-Aldosterone system (RAAS) overactivation and the expression of vascular endothelial growth factor protein (VEGF) and transforming growth factor-β1 (TGF-β1) (29). Earthworm protein capsule also has antihypertensive effects in humans (30). In the current study, one patient dropped out of the trail due to low blood pressure. This patient was simultaneously taking other traditional Chinese medicines that act on the cardiovascular system; therefore, it is unclear whether the low blood pressure was directly related to the trail material used in this study. However, because a drop in blood pressure is a common non-motor symptom in patients with PD, levodopa aggravates this tendency. To ensure safety, future studies should aim to evaluate the incidence and severity of hypotension in a larger cohort of subjects, which may provide a clearer understanding of the potential risks associated with the intervention and help establish appropriate safety guidelines.

## 5 Conclusion

The combination of probiotic *BF839* and earthworm protein as an adjunctive therapy for PD remarkably improved motor and some non-motor symptoms without serious adverse effects. The trial group demonstrated a substantial increase in *Enterococcus faecium* levels, while *Klebsiella* exhibited a notable decrease. These findings provide potential clues that may explain some of the underlying mechanisms. Further studies with larger sample sizes are warranted to more precisely define the efficacy, adverse effects, and underlying mechanisms involved.

### 5.1 Limitations

This is only a small sample size pilot trial, the results need to be validated in more samples. We did not employ specifically non-motor symptoms scales for PD patients to assess their cardiovascular symptoms, sleep and gastrointestinal function et al., which leading to the result is imperfection. We tested only 20 stool samples and can not exclude the possibility that maltodextrin could affect the gut microbiota, which resulted in insufficient confidence in the results of gut microbiota testing. We do not record the levodopa equivalent daily dose, also a limitation of the experiment.

## Data Availability

The original contributions presented in the study are included in the article/Supplementary material, further inquiries can be directed to the corresponding author/s.
