# Supplementary material for: Effect of BF839 + earthworm protein supplement on motor and some non-motor symptoms of Parkinson’s disease: a randomized clinical trial
Source: Front Neurol. 2024 Sep 6;15:1371791. doi: 10.3389/fneur.2024.1371791 (PMC11412884; doi:10.3389/fneur.2024.1371791)
Supplement: Supplementary file 1 [file Data_Sheet_1.doc]

Supplementary Material

Supplementary Data

**Table S1. Comparison of the response rate between the trail and placebo groups**

|  | per-protocol analysis n（%） | | | intention-to-treat analysis n（%） | | |
| --- | --- | --- | --- | --- | --- | --- |
| score decrease | trail | placebo | *P* | trail | placebo | *P* |
| UPDRS total≥8 points | 11/21  (52.38%） | 1/21  (4.77%) | <0.001 | 11/23（47.82%） | 1/23  (4.34%) | 0.001 |
| UPDRS part II≥2 points | 14/21  (66.67%) | 4/21  (19.05%) | 0.002 | 14/23  (60.87%) | 4/23  (17.39%) | 0.003 |
| UPDRS part III  ≥5 points | 7/21  (33.33%) | 1/21  (4.76%) | 0.018 | 7/23  (30.43%) | 1/23  (4.34%) | 0.020 |
| CSS  >30% | 20/21  (95.24%) | 0/19  (0%) | <0.001 | 22/23  (95.65%) | 0/23  (0%) | <0.001 |

*P*: Comparison between trail and placebo groups using chi-square test

## Outcome measures and follow-up

Assessments were performed at baseline, and at 6 and 12 weeks post-treatment. Participants underwent a face-to-face clinical assessment, performed at the same time of day in the hospital. Primary outcomes were measured using the United Parkinson's Disease Rate Scale (UPDRS) [1]. To clarify more symptomatic changes, more detailed score changes were also assessed, including UPDRS part I (Mentation, Behavior, Mood); UPDRS part II (Activities of daily living); UPDRS part III (Motor examination ), including UPDRS part III (Speed、Facial expression), UPDRS part III (Tremor at rest、action or Postural Tremor of hands), UPDRS part III (Rigidity), UPDRS part III (Figer Taps、Hand movements、Rapid Alternating Movements of Hands), UPDRS part III (Leg agility、Arising from Chair), UPDRS part III (Posture 、 Gait、Postural Stablility、Body Bradykinesia and Hypokinesia); and UPDRS part IV (Dyskinesias ). The secondary outcomes were measured using the Mini-Mental State Examination; Hamilton Depression Scale-24 (HAMD-24) [2]; Self-Rating Anxiety Scale (SAS) [3]; and Constipation Scoring System (CSS) [4]. The response rate was defined as the proportion of Minimal Clinically Important Difference change in patient scores from baseline to 12 weeks. For Hoehn and Yahr stages I–III, a change of eight, five, and two points were found to be the most appropriate cutoff for the UPDRS total, UPDRS part III, and UPDRS part II score, respectively [5]. Constipation response rate was defined as “30% decrease in CSS score [6]”.

We conduct follow-up phone calls every two weeks to remind participants to take their medication on schedule. During face-to-face consultations, which occur every 1.5 months, we collect the empty bags of consumed medication as a basis for calculating medication usage. All participants adhere strictly to the prescribed regimen. Furthermore, no participant has withdrawn from the study due to missing more than 20% of their prescribed doses.

## Production of BF839 and earthworm protein

1. BF839 is a live bacterial preparation obtained from well-developed infant intestinal flora and fermented in a self-prepared anaerobic fermentation medium. The composition of the anaerobic fermentation medium is: peptone 0.5-25%, glucose 0.2-0.5%, cysteine 0.01-0.5%, NaCl 0.2-0.5%, Na2HPO4 0.1-0.3%, sodium thioglycolate 0.02-0.1%, beef liver or pork liver extract 30-100%, beef extract 30-100%, yeast extract 0.1-0.8%. Fermentation is carried out at 32-40℃ for 12-72 hours, and the supernatant is centrifuged at 3500 rpm for bacterial application.

2)The production method of earthworm protein: (1). Cleaning: After the living earthworm spits out the food in its body, clean it; (2). Dissolving: Soak the cleaned living earthworm in a container of 40-50℃ warm water until it dies, remove it and place it in a container, add sodium bisulfite and mix evenly, and let it self-dissolve at room temperature for 10-48 hours; (3). Grinding: Grind the liquid with residual earthworm body after self-dissolution in step 2 to obtain a slurry; (4). Filtration and centrifugation: Filter the slurry obtained in step 3 with a 60-300 mesh sieve, take the filtrate, and centrifuge it at a speed of 5000-15000 r/min for 5-30 minutes with a centrifuge to remove the residue, obtaining the filtrate;(5) Membrane separation: First, use an ultrafiltration device to separate the filtrate from step 4 using an ultrafiltration membrane with a molecular weight cutoff of 9000-10000 Dalton to obtain the filtrate. Then, use an ultrafiltration device to separate the filtrate using an ultrafiltration membrane with a molecular weight cutoff of 8000 Dalton to remove the filtrate and obtain the concentrated solution for use. (6). Drying: Dry the concentrated solution directly to obtain the finished powder product, which is the active earthworm protein product.We didn't refine it any further

## Stool Sample collection

Stool samples were collected from 20 randomly selected patients on both day 0 and week 12. Ten patients were selected from the placebo group; two failed to provide stool samples at week 12, resulting in a total of 18 samples. In the test group, 10 patients were selected, resulting in a total of 20 samples. Stool samples were collected simultaneously at home using a sterile stool sample collection kit, followed by the addition of a DNA preservation solution. The samples were shipped to Shenzhen 01 Life Institute Co. Ltd. at room temperature for testing. Before sequencing, all samples were stored at -80°C.

## DNA extraction from stool samples and metagenomic sequencing

A 200 mg stool sample was mixed well with ethylenediaminetetraacetic acid (0.5 M, pH 8.0) and 550 uL lysis buffer, followed by the addition of Proteinase K and 20 μL lysozyme and incubation at 55 °C for 2 h. Subsequently, the mixture was centrifuged at 13,400 *g* for 5 minutes. The supernatant was then carefully aspirated, and 5 M sodium chloride (NaCl) was added. The mixture was gently vortexed for 10 s and allowed to stand at -20 °C for several minutes. Finally, the mixture was re-centrifuged at *13,400 g* for 10 min. The supernatant was aspirated into an Eppendorf (EP) tube, followed by centrifugation at 13,400 *g* for 5 min, supernatant aspiration into another new EP tube, isopropanol addition, allowing to stand at -20 °C for 20 min, and re-centrifugation at 13,400 *g* for 10 min. Subsequently, the liquid was added without removing any precipitate. Thereafter, the precipitate was washed twice with 1 mL 75% ethanol, with collection of the remaining liquid by centrifugation and aspiration using a pipette tip. To avoid over-drying the DNA sample, the EP tube was placed under a clean bench with the cap open for blow-drying. Finally, 50 uL double-distilled water (ddH2O) was added to dissolve the DNA sample, followed by the addition of 1 uL ribonuclease (RNase) A, sufficient mixing, and incubation at 37 °C for 15 min.

The degree of DNA degradation/contamination and total DNA amount were assessed via Qubit 2.0 using a 1% agarose gel. Samples with total DNA >1 ug and a brightness ratio of 1.8–2.0 were used for sequencing. The sequencing library was constructed by the NEBNext® Ultra™ DNA Library Prep Kit; moreover, an indexed sequence was added for each sample. The generated DNA library was sequenced on an Illumina high-throughput sequencing platform. The libraries were constructed as follows: DNA was first fragmented to approximately 350 bp using ultrasound, followed by end repair, A-tail addition, and addition of Illumina adapters through polymerase chain reaction (PCR). PCR products were purified using the AMPure XP system. To ensure library quality, the distribution of library fragment lengths was determined using an Agilent 2100, and the effective library concentration was determined through quantitative PCR.

## DNA quality control

Thirty-eight samples from 20 patients were sequenced, and raw base data from 253.32 Gbp were generated. Quality control was performed using sequencing data quality control software (fastq_trim_filter_v5_EMBL) in Trimmomatic software, where (1) adapter sequences and sequences with >3 N bases in a single sequence were removed, and (2) low-quality sequences were removed using length and quality thresholds of 30 bp and 20 bp, respectively. The sequencing data were aligned with the human genome GRCh38 using the alignment tool SOAP2 (version 2.20); moreover, host contamination was removed with a similarity threshold of 90%. A total of 249.35 Gbp (cleaned data) were retained in the dataset for downstream analysis.

## Alignment with reference gene set and intestinal metabolic module prediction

Gene set alignment and calculation of relative abundance were performed as previously described [7], and gene coding-related intestinal metabolic modules were predicted using a module-based analysis method, as previously described [8], along with MetaCyc, a metabolism database. Briefly, SOAP2 (version 2.20) was used to align the sequencing data with the reference gene set to filter the best-aligned sequences among those with >90% similarity for the final alignment result. Relative gene abundance was calculated as previously described. Relative abundances in the Kyoto Encyclopedia of Genes and Genomes Orthology (KO) database were obtained by summing and normalizing the relative abundances of annotated genes. The abundance of intestinal metabolic modules was calculated from combinations in the KO database comprising intestinal metabolic modules.

## Species annotation

Species annotation was performed using MetaPhlAn (default parameters), and abundance was calculated at the phylum, genus, and species levels.

## Bioinformatics analysis

Bioinformatics analyses were performed using R3.6.3. The observed number of species (count) and Shannon diversity of the samples were calculated using the species-level abundance file of MetaPhlAn to assess changes in microbiota diversity in the fecal samples. The Bray-Curtis distance similarity matrix was calculated using the species-level abundance file of MetaPhlAn, followed by principal coordinate analysis (PCoA) to determine microbiota composition changes. Additionally, we performed a permutational multivariate analysis of variance (999 permutation tests) using the vegan R package to determine differences in microbiota composition over time and according to group.

# References

[1] S. Fahn, C.D. Marsden, D. Calne, Recent Developments in Parkinson’s Disease, MacMillan, Florham Park, New Jersey, pp. 153–164, 1987.

[2] M. Hamilton, A rating scale for depression, J. Neurol. Neurosurg. Psychiatry. 23 (1960) 56–62. https://doi.org/10.1136/jnnp.23.1.56, http://www.ncbi.nlm.nih.gov/pubmed/14399272, 495331.

[3] W.W. Zung, A rating instrument for anxiety disorders, Psychosomatics. 12 (1971) 371–379. https://doi.org/10.1016/S0033-3182(71)71479-0, http://www.ncbi.nlm.nih.gov/pubmed/5172928.

[4] F. Agachan, T. Chen, J. Pfeifer, P. Reissman, S.D. Wexner, A constipation scoring system to simplify evaluation and management of constipated patients, Dis. Colon Rectum. 39 (1996) 681–685. https://doi.org/10.1007/BF02056950, http://www.ncbi.nlm.nih.gov/pubmed/8646957.

[5] A. Schrag, C. Sampaio, N. Counsell, W. Poewe, Minimal clinically important change on the unified Parkinson’s disease rating scale, Mov. Disord. 21 (2006) 1200–1207. https://doi.org/10.1002/mds.20914, http://www.ncbi.nlm.nih.gov/pubmed/16673410.

[6] S. Hong, Z. Lu, Y. Bai, Expert consensus on TCM diagnosis and treatment of constipation, China Med. 36 (2017). Beijing Trad 771–776 + 784. https://doi.org/10.16025/j.1674-1307.2017.09.001 (in Chinese).

[7] J. Qin, Y. Li, Z. Cai, S. Li, J. Zhu, F. Zhang, S. Liang, W. Zhang, Y. Guan, D. Shen, Y. Peng, D. Zhang, Z. Jie, W. Wu, Y. Qin, W. Xue, J. Li, L. Han, D. Lu, P. Wu, Y. Dai, X. Sun, Z. Li, A. Tang, S. Zhong, X. Li, W. Chen, R. Xu, M. Wang, Q. Feng, M. Gong, J. Yu, Y. Zhang, M. Zhang, T. Hansen, G. Sanchez, J. Raes, G. Falony, S. Okuda, M. Almeida, E. Lechatelier, P. Renault, N. Pons, J.M. Batto, Z. Zhang, H. Chen, R. Yang, W. Zheng, S. Li, H. Yang, J. Wang, S.D. Ehrlich, R. Nielsen, O. Pedersen, K. Kristiansen, J. Wang, A metagenome-wide association study of gut microbiota in type 2 diabetes, Nature. 490 (2012) 55–60. https://doi.org/10.1038/nature11450, http://www.ncbi.nlm.nih.gov/pubmed/23023125.

[8] M. Valles-Colomer, G. Falony, Y. Darzi, E.F. Tigchelaar, J. Wang, R.Y. Tito, C. Schiweck, A. Kurilshikov, M. Joossens, C. Wijmenga, S. Claes, L. Van Oudenhove, A. Zhernakova, S. Vieira-Silva, J. Raes, The neuroactive potential of the human gut microbiota in quality of life and depression, Nat. Microbiol. 4 (2019) 623–632. https://doi.org/10.1038/s41564-018-0337-x, <http://www.ncbi.nlm.nih.gov/pubmed/30718848>.

1. **The packaging for the placebo group and the experimental group.**


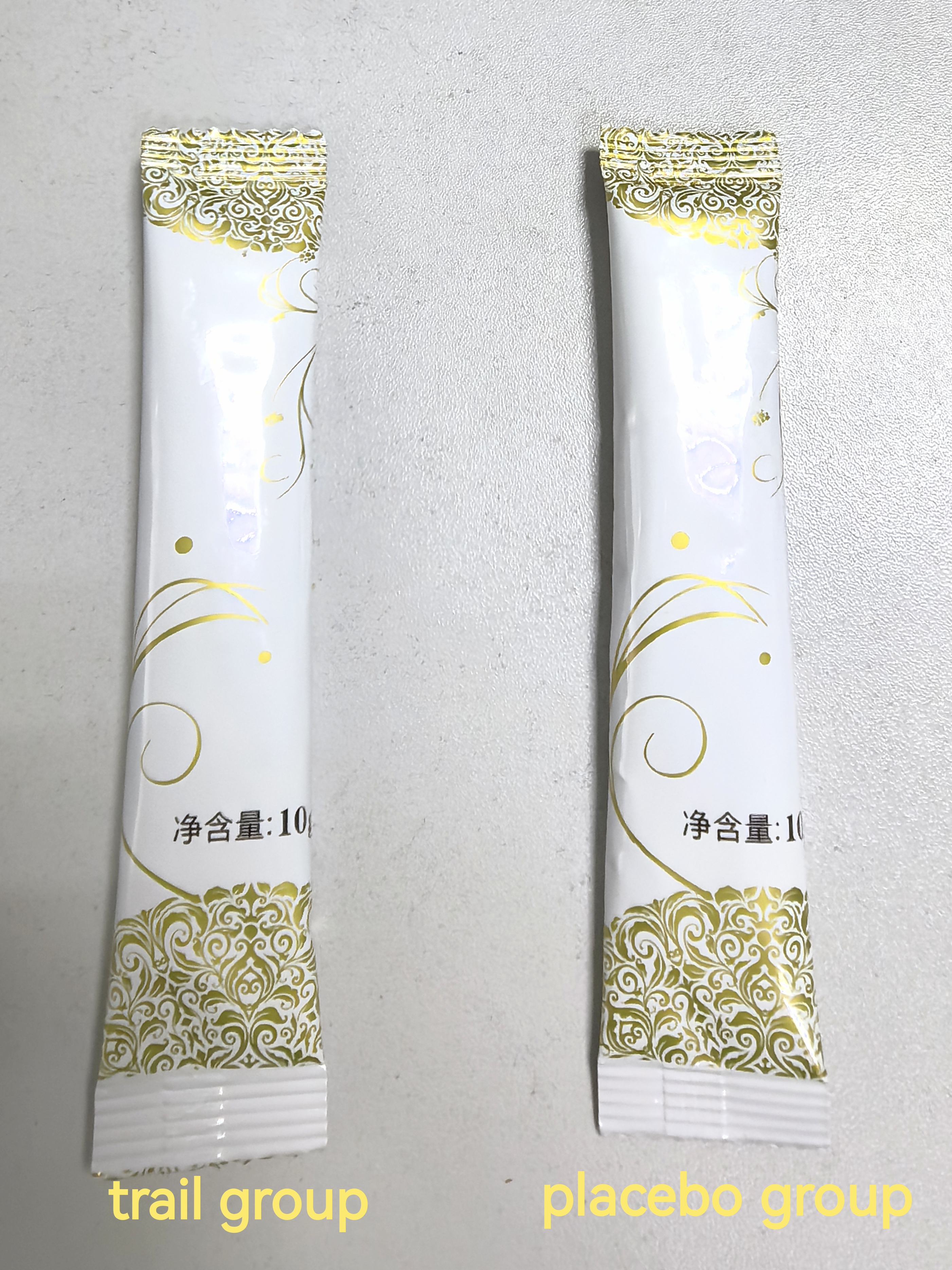


1. **The sample size estimation formula.**


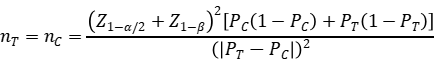


*P*_T_ and *P*_C_ are the expected event rates of the test group and control group, respectively

αset 0.05，
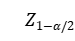
=1.9599639845；

βset 0.2，
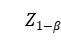
=0.8416212336；
